# Clinical Evaluation of an Innovative CaHA Bio‐Stimulator in Facial Rejuvenation: 1 Year Follow‐Up

**DOI:** 10.1111/jocd.70210

**Published:** 2025-05-09

**Authors:** Giseli Petrone, Caroline Leão, Danilo Morais, Eliza Ducati, Matheus Kasai, Renata Viana

**Affiliations:** ^1^ Independent Researcher Rio de Janeiro Brazil; ^2^ Independent Researcher São Paulo Brazil; ^3^ Independent Researcher Minas Gerais Brazil; ^4^ Blanc Hospital São Paulo Brazil

**Keywords:** calcium hydroxilapatite, patient satisfaction, skin laxity

## Abstract

**Background:**

Skin aging is characterized by wrinkles, loss of elasticity, laxity, and rough texture, resulting from degenerative changes in the skin's layers. Effective treatments targeting skin structure are increasingly sought after in aesthetic medicine.

**Aims:**

This study aimed to evaluate the safety, efficacy, and long‐term outcomes of STIIM, a novel calcium hydroxylapatite (CaHA)‐based bio‐stimulator, in facial rejuvenation.

**Patients/Methods:**

Thirty‐two patients aged 35–59 received diluted CaHA injections. Outcomes were assessed through patient satisfaction surveys and objective measurements, including ultrasound evaluation of dermal thickness. Follow‐ups were conducted at 120 and 360 days post‐procedure. Safety was evaluated by monitoring adverse events.

**Results:**

At 120 days post‐treatment, 85% of patients reported visible aesthetic improvements, and ultrasound assessments in a subset of patients demonstrated a significant 51% increase in dermal thickness, indicating improved skin structure. At 360 days, 75% of the patients continued to report aesthetic benefits, confirming the durability of the results. The treatment was well‐tolerated, with no serious adverse events reported throughout the study period.

**Conclusions:**

This novel CaHA‐based bio‐stimulator demonstrates a safe and effective profile for facial rejuvenation, providing significant aesthetic and structural improvements with long‐lasting results. It offers a promising option for addressing the challenges of skin aging.

## 1 Introduction

Skin aging is characterized by features such as wrinkling, loss of elasticity, laxity, and a rough‐textured appearance due to degenerative changes in the skin's three layers: the epidermis, dermis, and subcutaneous tissue [1]. As the skin ages, these changes become more pronounced, leading to the development of fine wrinkles and thinning of the epidermis, which progresses to deeper wrinkles, increased skin laxity, and hyperpigmentation. Typical phenomena of skin aging are reduced elasticity and progressive atrophy of the dermis, largely attributed to a decrease in collagen production and an increase in its degradation [2].

In 2022, approximately 23.67 million cosmetic minimally invasive procedures were performed in the US, reflecting a significant patient interest and demand for aesthetic treatments [3]. Treatments such as fillers, autologous fat grafting, threads, bio‐stimulators, surgical methods, and technologies for skin resurfacing and skin tightening each offer distinct advantages and limitations, catering to diverse patient needs and preferences [4, 5, 6, 7, 8].

Among the available options for facial rejuvenation procedures, calcium hydroxylapatite (CaHA) is a well‐known bio‐stimulator in aesthetic medicine [9]. CaHA is a safe and effective substance that has been proven to stimulate collagen production [9], enhancing skin quality and appearance [10], with a favorable safety profile and a low incidence of adverse events [11].

This study evaluates the effects of STIIM, a novel CaHA‐based bio‐stimulator, on patient satisfaction, ultrasonographic outcomes, safety, and overall clinical results.

## 2 Materials and Methods

The study was approved by the ethics committee, and all participants provided written informed consent in accordance with the ethical guidelines of the Declaration of Helsinki 1975. Patients also consented to allow image use.

Clinical, demographic, and imaging data were collected on 32 patients sequentially submitted to the application of diluted CaHA in the face. Patients were also invited to complete a Global Aesthetic Improvement Scale (GAIS) and a satisfaction questionnaire.

### 2.1 Selection Criteria

Inclusion criteria included healthy adults aged 35 to 60 years old, scheduled to receive CaHA treatment for Facial Laxity (STIIM, diluted 1:1) at the clinic. Patients treated within 3 months of the visit were also invited to participate, and data was generated retrospectively.

Exclusion criteria included patients treated with a different dilution, those who underwent any facial rejuvenation procedures in the last 6 months, those who declined participation, and patients treated with a different brand of CaHA‐based bio‐stimulator.

A subgroup of patients was invited to participate in the imaging subgroup. Participants provided consent for ultrasound imaging immediately before the procedure and at the 3‐month follow‐up.

### 2.2 Material and Procedure

Calcium Hydroxilapatite bio‐stimulator STIIM (Ilikia, manufactured by CGBIO, South Korea), pre‐filled syringe was used. The product is composed of 30% CaHA and 70% carboxymethylcellulose gel, available in 1.5 mL syringes. It contains CaHA microspheres measuring 25–45 μm and features a lattice‐pore structure. According to the manufacturer, this structure creates a scaffold that is large, firm, and stable, contributing to long‐term efficacy. In addition, due to this specific structure, the particles degrade more slowly, resulting in prolonged clinical effects.

A total of two diluted syringes per patient were used at a dilution rate of 1:1 (1.5 mL CaHA, 1 mL of saline solution and 0.5 mL of 2% lidocaine with vasoconstrictor).

**FIGURE 1:**
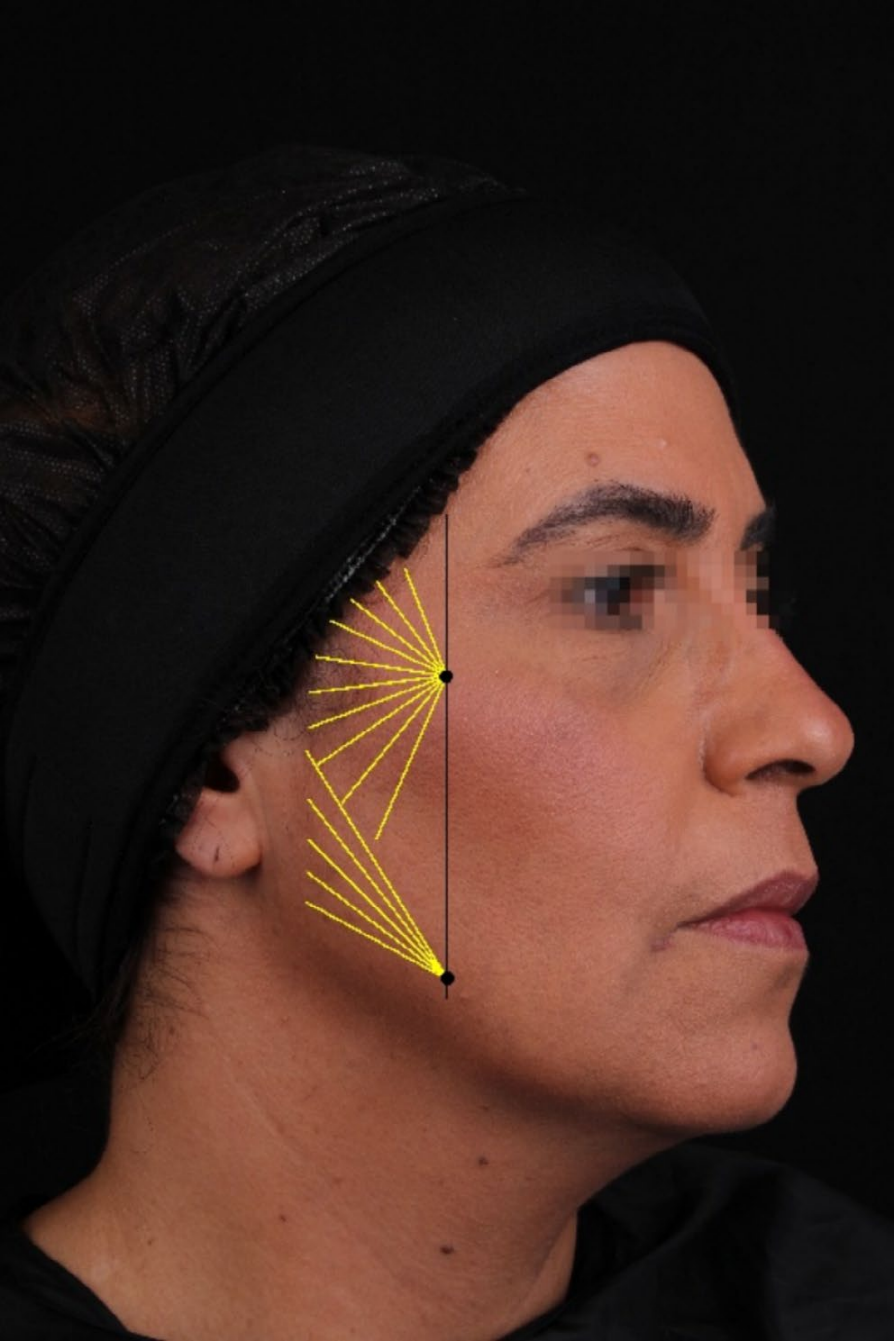
Ligament line (in black) and fan‐shaped distribution pattern (in yellow) for the application of the CaHA biostimulator on the face.

Patients received 1.5 mL of diluted CaHA per hemi‐face, applied into the superficial subcutaneous tissue using a 22G × 50 mm blunt‐tip cannula, in a fan‐shaped and retrograde distribution technique. Per clinical practice, the injections were made into the retro‐ligamentar area. Entry points were located on the zygoma to address the temporal and zygomatic areas up to the scalp insertion. Additionally, an entry point in the jowl region was made to target the infra‐zygomatic, pre‐auricular, and mandibular areas, as depicted in Figure 1. All patients received a gentle massage after application.

### 2.3 Data Collection

Demographics and clinical health data were collected, including age, height, weight, and relevant medical history, encompassing pre‐existing medical conditions and continuous medication use. Additionally, information regarding known allergies, previous facial‐related procedures, and ongoing use of facial cosmetics was recorded. Procedure data, such as the utilized dilution, application method, and treatment plan, were also gathered.

### 2.4 Photographic Imaging—Conventional and Three‐Dimensional

Access was requested to the photographic images routinely taken in the clinic of patients who agreed and signed the consent form for viewing and use of the images. Conventional 2D photography and 3D images (Quantificare LifeViz) were collected at the following time points: pre‐procedure, 120 and 360 days follow‐up.

### 2.5 Scales and Satisfaction Assessment

GAIS [12] was assessed by both the patient and an independent physician. Additionally, a supplementary patient satisfaction questionnaire was collected, incorporating a 5‐point Likert scale with parameters ranging from “completely dissatisfied” to “completely satisfied” at 120‐ and 360 days post‐procedure.

Furthermore, pre‐and post‐procedure (120 days) assessments by an independent physician using the 9‐class modified Facial Laxity Scale were collected [13].

### 2.6 Ultrasound Imaging

A subgroup of patients underwent ultrasound imaging using a high‐frequency ultrasound machine Logiq e (GE Healthcare, Chicago, IL, USA) set to a frequency of 9 to 22 MHz. This imaging aimed to evaluate the presence and distribution of the product, measure the thickness of the epidermis and dermis, and assess the transition and subcutaneous layers.

The ultrasound evaluations were conducted in a subset of patients at three time points: pre‐procedure, immediately post‐procedure, and 120 days post‐procedure. Images were analyzed to determine the precise location of the bio‐stimulator and measure changes in tissue thickness and structure over time.

### 2.7 Adverse Event Monitoring

Throughout the study period, continuous monitoring of adverse events was conducted. All adverse events reported by study participants were captured.

## 3 Results

### 3.1 Population Characteristics

The study population comprised 32 participants, with ages ranging from 35 to 59 years and an average age of 46.38 years.

There were 4 men and 28 women, indicating a predominance of female patients. The average body mass index (BMI) was 25.22 kg/m^2^. Most participants reported continuous use of moisturizing and sunscreen products (81.25% and 53.13%, respectively), highlighting their commitment to skincare and sun protection. Clinical data revealed that only 4 participants had undergone a previous facial rejuvenation procedure (12.50%). None of the patients had botulinic toxin application in the past 3 months, nor bio‐stimulator procedures in the previous 9 months from the baseline study procedure. Clinical demographics and characteristics are displayed in Table 1.

**TABLE 1:** Demographic and clinical data of study participants.

Characteristic		Total (*n* = 32)
Gender		
Female		28 (88%)
Male		4 (12%)
Age (years)		
Mean		46.38
SD		6.35
BMI (kg/m²)		
Mean		25.22
SD		3.87
Phototype		
Phototype I		1 (3.13%)
Phototype II		5 (15.63%)
Phototype III		16 (50%)
Phototype IV		9 (28.13%)
Phototype V		1 (3.13%)
Clinical evaluation		
Acne		1 (3.13%)
Melasma		11 (34.38%)
Rosacea		5 (15.63%)
Solar melanosis		14 (43.75%)
Skin care habits		
Daily sunscreen		26 (81.25%)
Daily facial moisturizer		17 (53.13%)
Previous aesthetic procedure		
Yes		4 (12.50%)
Facial laxity scale – class		
Mild	1	1 (3%)
	2	2 (6%)
	3	5 (16%)
Moderate	4	0 (0%)
	5	10 (31%)
	6	3 (9%)
Severe	7	3 (9%)
	8	3 (9%)
	9	5 (16%)

### 3.2 Clinical Outcomes

The GAIS assessment of pre‐ and post‐procedure images was rated by a medical researcher. A total of 81% of the patients were rated as “Very Much Improved,” “Much Improved,” or “Improved” at 120 days endpoint.

Important improvement in facial laxity was demonstrated through the Facial Laxity Scale rating, with 71.43% of patients demonstrating at least a one‐point reduction on the scale at 120‐day timepoint. The average improvement was −1.6 points on the scale, indicating a reduction in facial laxity.

**FIGURE 2:**
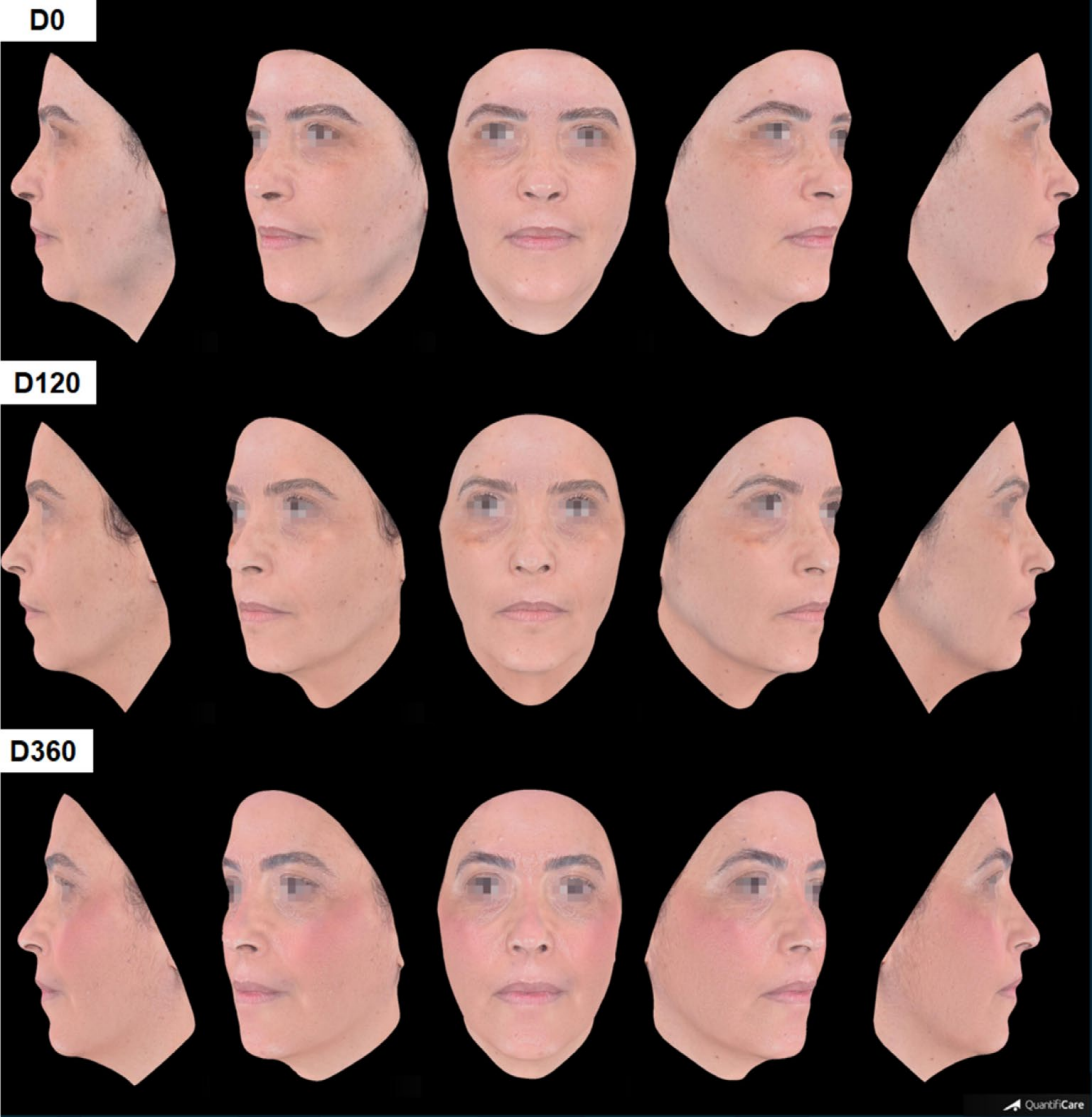
Three‐dimensional photographic evaluations of a 48‐year‐old patient pre (D0), 120‐ and 360‐days post treatment.

**FIGURE 3:**
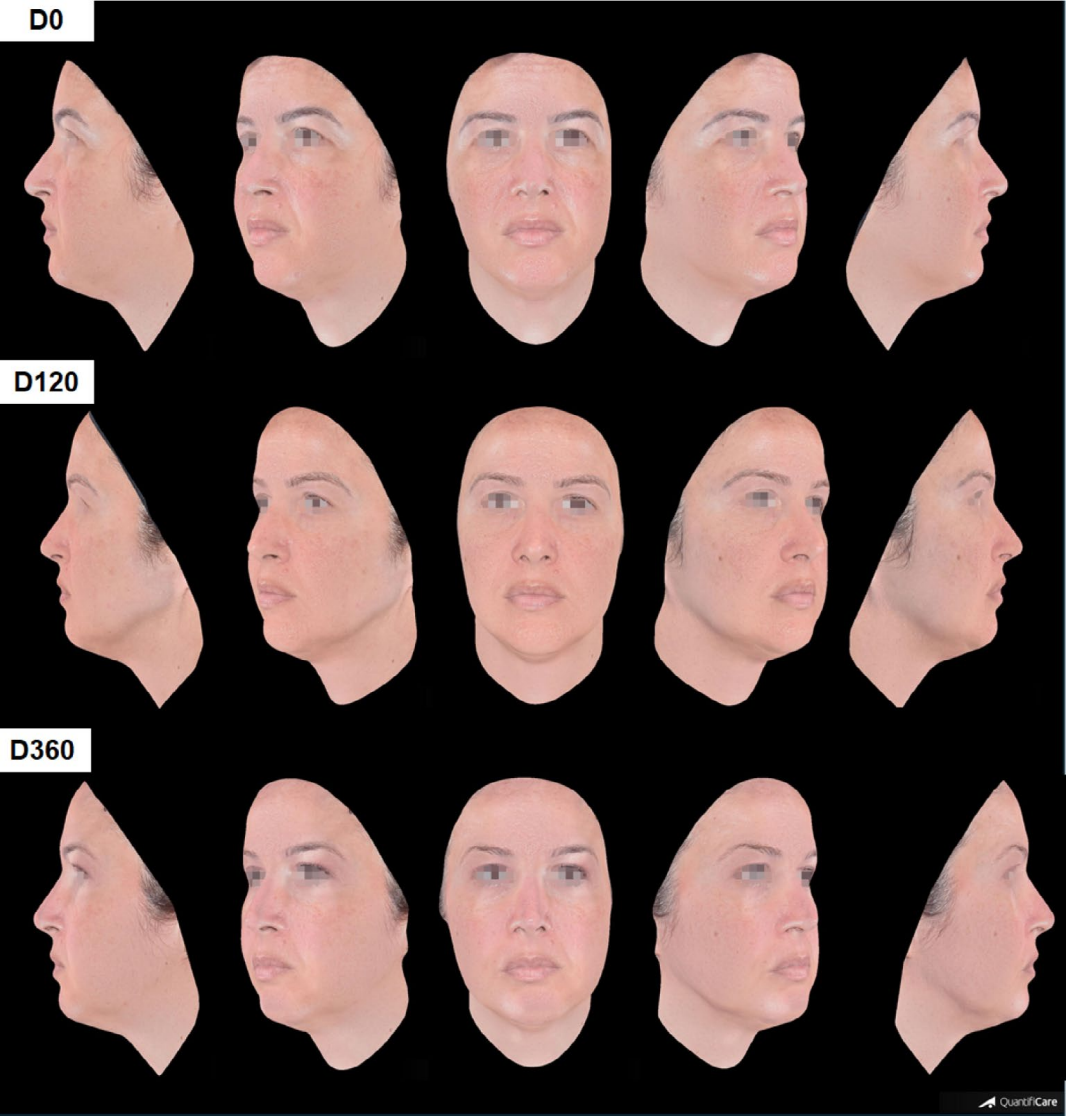
Three‐dimensional photographic evaluations of a 40‐year‐old patient pre (D0), 120‐ and 360‐days post treatment.

**FIGURE 4:**
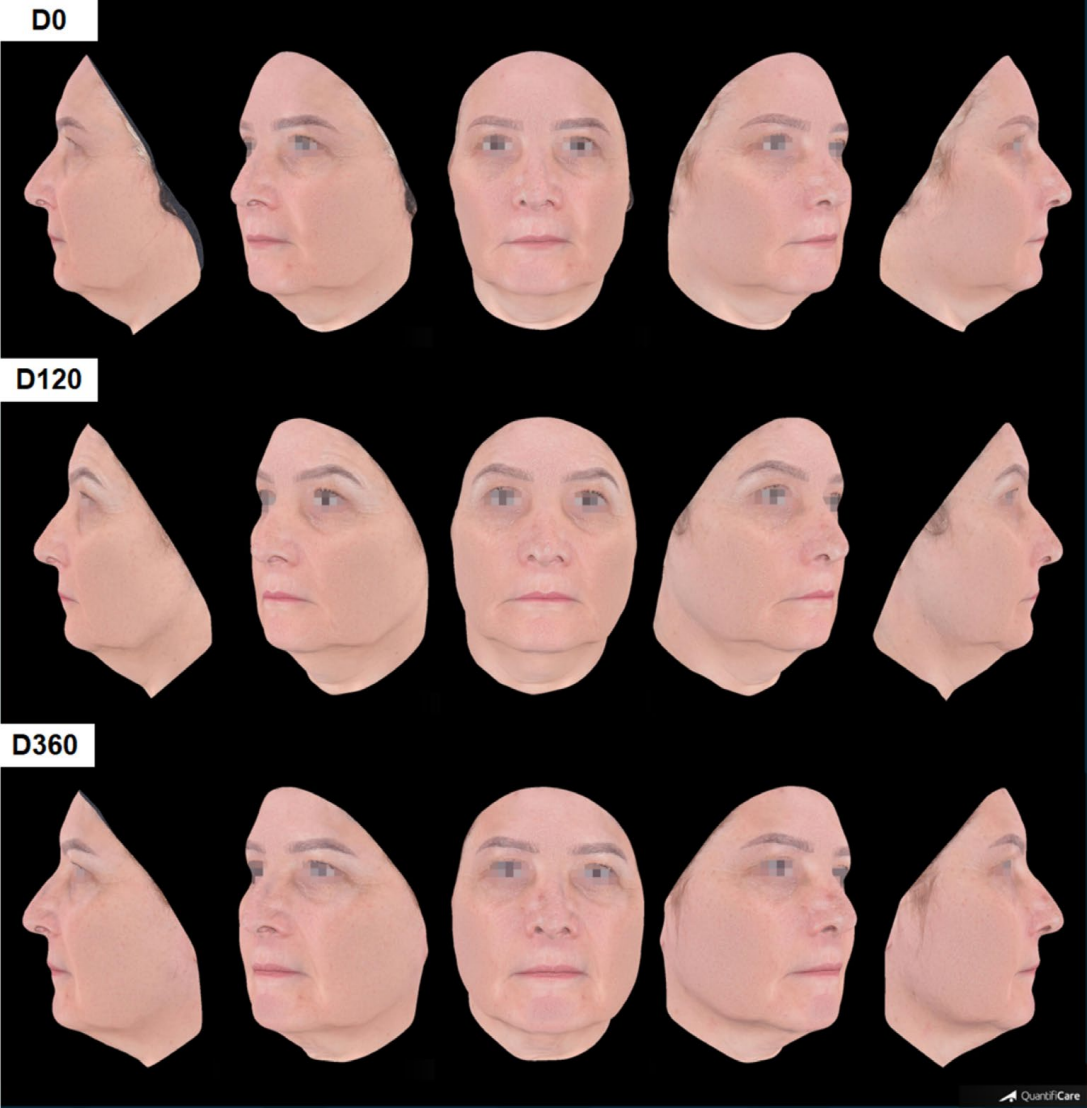
Three‐dimensional photographic evaluations of a 57‐year‐old patient pre (D0), 120‐ and 360‐days post treatment.

Clinical outcome through photographic images is illustrated in Figures 2, 3, 4. Improvement in other clinical aspects, such as melasma, melanosis, rosacea, and acne, was also observed and reported by the physician during evaluation of pre‐ and 120‐day photographic images, although it was not rated. These images capture detailed changes, improvements in skin texture, and facial contour following treatment.

### 3.3 Patient Satisfaction

**FIGURE 5:**
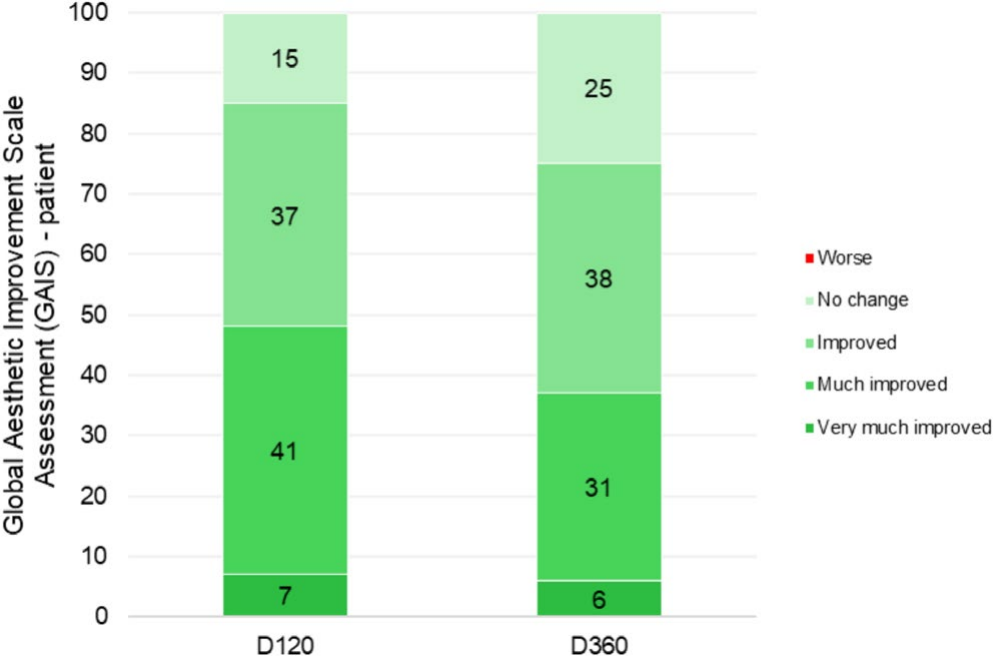
Patient‐Self‐Assessed Global Aesthetic Improvement Scale (GAIS). This graph presents the distribution of patients based on their own self‐assessments using the GAIS 120 and 360 days after the procedure.

High patient satisfaction was achieved in the GAIS at 120 days post‐procedure (Figure 5). According to GAIS, 85% of patients reported aesthetic facial improvement, where 7% of patients rated GAIS as “Very Much Improved,” 41% as “Much Improved,” and 37% as “Improved” at 120 days. Among the patients who rated “No Change” on GAIS, half of those were classified as overweight.

At 360 days post‐procedure, we were able to contact only 16 patients for follow‐up. Among these, 75% reported some degree of improvement. Specifically, 6% rated their condition as “Very Much Improved,” 31% as “Much Improved,” and 38% as “Improved.” Meanwhile, 25% reported “No Change,” and no patients indicated a worsening of their condition.

Prior to the procedure (D0), 78% of patients reported dissatisfaction with the overall appearance of the face, with 9% describing themselves as “Completely Dissatisfied” and 69% as “Dissatisfied.” However, 120 days after the procedure, only 4% of patients expressed dissatisfaction, showing an expressive patient satisfaction with the results. By 360 days post‐procedure, among the 16 patients evaluated, 19% reported dissatisfaction. Detailed data are summarized in Table 2.

**TABLE 2:** Patient satisfaction levels using the 5‐point scale.

	Very satisfied	Satisfied	Neutral	Dissatisfied	Very dissatisfied
D0	0%	16%	6%	69%	9%
D120	7%	78%	11%	4%	0%
D360	25%	31%	25%	19%	0%

*Note:* This table shows the distribution of patient satisfaction levels as assessed by the 5‐point scale, at pre‐procedure (D0) and 120 and 360 days after the procedure.

### 3.4 Ultrasound Imaging Results

**FIGURE 6:**
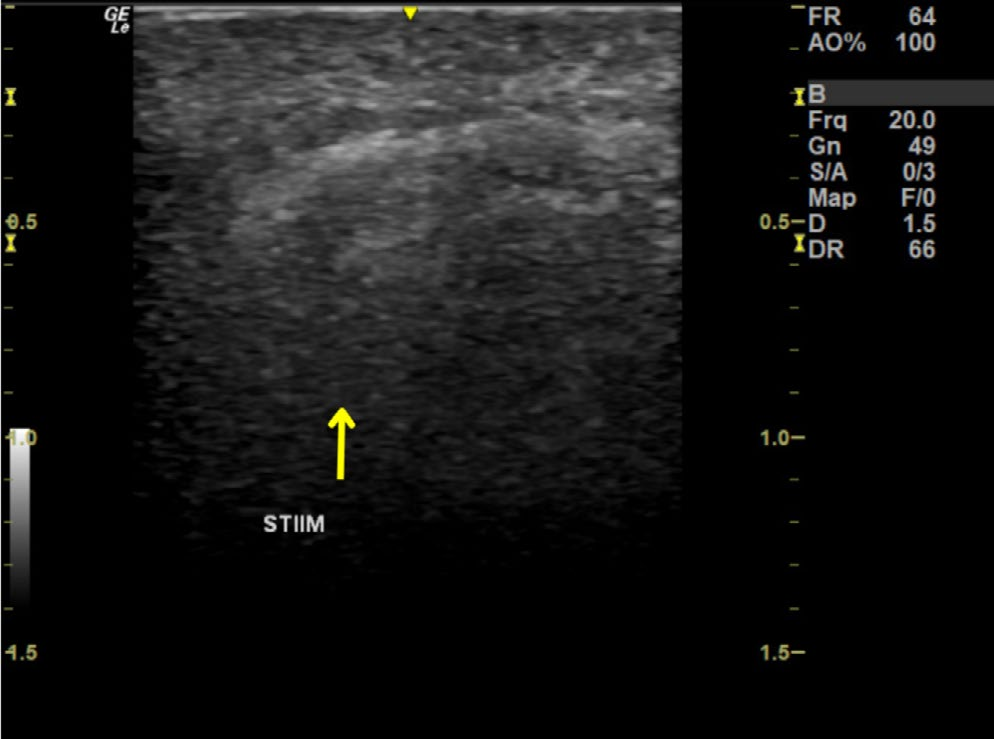
Immediate post‐procedure ultrasound image showing the distribution of the CaHA‐based biostimulator (STIIM) within the superficial subcutaneous layer. The hyperechoic areas, indicated by the arrow, correspond to the calcium hydroxyapatite biostimulator, demonstrating its uniform distribution within the tissue.

Ultrasound imaging was obtained to assess the distribution and integration of the CaHA‐based biostimulator (STIIM) within the subcutaneous tissue, immediately post‐procedure. Figure 6 demonstrates the hyperechoic areas, indicated by an arrow, which correspond to the STIIM particles. This imaging shows a uniform dispersion of the product without clustering.

**FIGURE 7:**
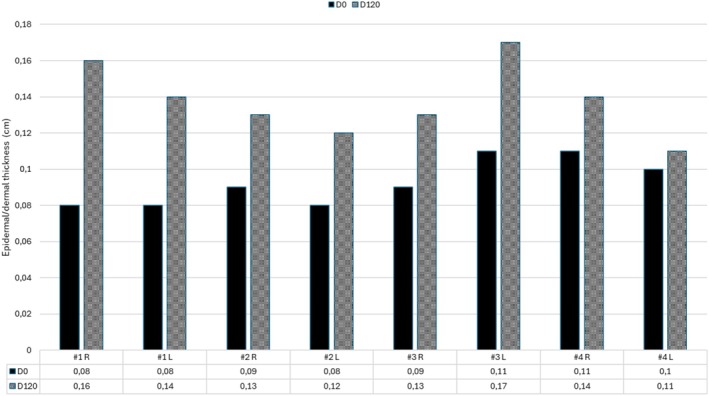
Epidermal/dermal thickness pre‐procedure and at 120 days after treatment.

Ultrasound imaging to evaluate the effects of the treatment in the dermis was assessed pre and 120 days post‐procedure in a subset of patients. The mean age of the patients in this subgroup was 46.75 years. The results demonstrated that all 4 evaluated patients experienced a significant increase in epidermal/dermal thickness 120 days after the procedure, with an average increase of 51% thickness. Detailed data on epidermal/dermal thickness increase are presented in the graph in Figure 7. At 360 days, the ultrasound imaging was not performed due to the unavailability of the patients on the scheduled dates.

### 3.5 Adverse Event Monitoring

No serious adverse events were reported. Minimal and limited local expected reactions were captured post‐procedure, as 4 patients reported bruising, 6 patients experienced light pain upon touch, and 2 patients had mild edema. All reactions were self‐limited and resolved spontaneously within 15 days.

## 4 Discussion

Significant improvements were observed in clinical evaluations and patient satisfaction metrics supporting the application of the product in aesthetic procedures.

Clinical outcomes measured by the GAIS reflect high patient satisfaction, and the Facial Laxity Scale reflects its efficacy in reducing laxity and improving overall facial aesthetics. At 120 days post‐procedure, a notable percentage of patients showed improvement in both GAIS and the laxity scale. These findings align with existing literature on CaHA‐based biostimulators, which have been shown to provide durable and natural‐looking results. A systematic review by Amiri et al. (2024) included 13 studies and found that CaHA injections were associated with significant improvements in the GAIS, high patient satisfaction in facial treatments, and a reduction in facial wrinkles [14]. Other studies observed notable and long‐lasting improvements in facial appearance with repeated CaHA injections [15] and high satisfaction rates for treatments of mid‐face volume loss with CaHA [16].

At 360 days post‐procedure, 75% of patients still reported aesthetic facial improvement on the GAIS, demonstrating long‐lasting outcomes and patient satisfaction.

Ultrasound imaging of a subset of patients revealed a marked increase in epidermal and dermal thickness, averaging a 51% increase at 120 days post‐procedure. This is a relevant finding as it demonstrates the effectiveness of CaHA‐based bio‐stimulators in promoting significant improvements in skin thickness and overall dermal structure. This data is corroborated by the findings in an animal in vivo study [17] which demonstrated that CaHA/CMC gel leads to a significant increase in dermal thickness, elastic fibers, and collagen density. It is also demonstrated by another clinical study of a combined dermal filler (hyaluronic acid and CaHA), showing a significant increase in dermal thickness at 90 and 120 days [17].

Although the product was injected into the superficial subcutaneous plane, the increase in dermal thickness observed at 120 days may be attributed to the biostimulatory effects of CaHA on dermal remodeling. A review of the literature shows that the specific effects of CaHA in each anatomical layer are not yet fully elucidated. Yutskovskaya and Kogan [18] reported a statistically significant increase in dermal thickness assessed by ultrasound (from 1462.3 to 1865.9 μm) following treatment with diluted CaHA, along with enhanced expression of collagen types I and III and elastin in histological evaluation. In that study, as in ours, subcutaneous thickness was not evaluated. However, no clinical signs or ultrasound findings suggestive of fibrosis or subcutaneous volume reduction were observed during follow‐up. Future studies may help clarify the remodeling dynamics in different skin layers.

The safety profile is consistent with the literature as no serious adverse events were reported in this study during the 360‐day follow‐up period. In a review, including 21 studies involving 2779 patients and 5081 CaHA treatments, 3% of adverse events were reported. The majority of the reported events were nodules (96%), and a minority showed persistent inflammation/swelling (2%), persistent erythema (1%), and overcorrection (1%) [11]. Moreover, clinical trials following patients for up to 3 years post‐injection reported no long‐term or delayed‐onset adverse events [19].

A limitation of this study is observed as having a small sample size, a lack of a control group, and lost to follow‐up patients at 360 days. Future studies with larger cohorts, comparative groups, and improved follow‐up rates are necessary to validate these findings and further establish STIIM's comparative effectiveness against other bio‐stimulators.

## 5 Conclusion

The study highlights the promising clinical outcomes, high patient satisfaction, and favorable safety profile associated with STIIM for facial rejuvenation. This CaHA‐based bio‐stimulator appears to offer significant benefits in improving facial aesthetics. Further research, particularly studies involving direct comparisons with other products, will be instrumental in establishing STIIM's role in clinical practice.

## Author Contributions

All authors (G.P., C.L., D.M., E.D., M.K., R.V.) contributed to the conception and design of the study, as well as to the analysis and interpretation of the data. R.V. and G.P. were responsible for drafting the initial version of the manuscript. All authors (G.P., C.L., D.M., E.D., M.K., R.V.) critically revised the manuscript and approved the final submitted version.

## Ethics Statement

This study was conducted in accordance with the principles of the Declaration of Helsinki and approved by the Ethics Committee under approval number 6.537.883.

## Conflicts of Interest

M.K., D.M., and C.L. provide lectures and training on behalf of Ilikia Brasil, the distributor of the product used in this study. R.V. serves as a scientific consultant for Ilikia Brasil. G.P. previously delivered lectures for Ilikia Brasil but currently has no affiliation with the company. E.D., who was responsible for the ultrasound analyses in the study, declares no conflicts of interest.

## Data Availability

The data that support the findings of this study are available from the corresponding author upon reasonable request.

## References

[1] S. Zhang and E. Duan , “Fighting Against Skin Aging,” Cell Transplantation 27, no. 5 (2018): 729–738. 29692196 10.1177/0963689717725755 PMC6047276

[2] J. W. Shin , S. H. Kwon , J. Y. Choi , et al., “Molecular Mechanisms of Dermal Aging and Antiaging Approaches,” International Journal of Molecular Sciences 20, no. 9 (2019): 2126. 31036793 10.3390/ijms20092126 PMC6540032

[3] American Society of Plastic Surgeons , “The American Society of Plastic Surgeons Procedural Statistics Data Insights,” 2023. 10.1097/01.PRS.0000084284.02024.3B 14504525

[4] D. H. Jones , “Semipermanent and Permanent Injectable Fillers,” Dermatologic Clinics 27, no. 4 (2009): 433–444. 19850193 10.1016/j.det.2009.08.003

[5] R. F. Mazzola , G. Cantarella , S. Torretta , A. Sbarbati , L. Lazzari , and L. Pignataro , “Autologous Fat Injection to Face and Neck: From Soft Tissue Augmentation to Regenerative Medicine,” Acta Otorhinolaryngologica Italica 31, no. 2 (2011): 59–69. 22058586 PMC3203738

[6] P. Irina and K. Albina , “Single‐Blind Comparative Study of the Aesthetic Outcome of Armouring Procedures With PLLA/PCL‐ and HA‐Enriched Absorbable Threads,” Trichology and Cosmetology—Open Journal 3, no. 1 (2018): 15–20, https://openventio.org/wp‐content/uploads/Single‐Blind‐Comparative‐Study‐of‐the‐Aesthetic‐Outcome‐of‐Armouring‐Procedures‐with‐PLLA‐PCL‐and‐HA‐enriched‐Absorbable‐Threads‐TCOJ‐3‐113.pdf.

[7] M. H. Graivier , L. S. Bass , M. Busso , M. E. Jasin , R. S. Narins , and T. L. Tzikas , “Calcium Hydroxylapatite (Radiesse) for Correction of the Mid‐ and Lower Face: Consensus Recommendations,” Plastic and Reconstructive Surgery 120 (2007): 55S–66S. 18090343 10.1097/01.prs.0000285109.34527.b9

[8] W. Dou , Q. Yang , Y. Yin , et al., “Fractional Microneedle Radiofrequency Device and Fractional Erbium‐Doped Glass 1565‐Nm Device Treatment of Human Facial Photoaging: A Prospective, Split‐Face, Random Clinical Trial,” Journal of Cosmetic and Laser Therapy 23, no. 5–6 (2021): 142–148, 10.1080/14764172.2022.2033783. 35083965

[9] B. Nowag , G. Casabona , S. Kippenberger , N. Zöller , and T. Hengl , “Calcium Hydroxylapatite Microspheres Activate Fibroblasts Through Direct Contact to Stimulate Neocollagenesis,” Journal of Cosmetic Dermatology 22, no. 2 (2023): 426–432. 36575882 10.1111/jocd.15521

[10] A. T. de Almeida , V. Figueredo , A. L. G. da Cunha , et al., “Consensus Recommendations for the Use of Hyperdiluted Calcium Hydroxyapatite (Radiesse) as a Face and Body Biostimulatory Agent,” Plastic and Reconstructive Surgery. Global Open 7, no. 3 (2019): e2160. 31044123 10.1097/GOX.0000000000002160 PMC6467620

[11] J. A. Kadouch , “Calcium Hydroxylapatite: A Review on Safety and Complications,” Journal of Cosmetic Dermatology 16 (2017): 152–161. 28247924 10.1111/jocd.12326

[12] R. S. Narins , F. Brandt , J. Leyden , P. Z. Lorenc , M. Rubin , and S. Smith , “A Randomized, Double‐Blind, Multicenter Comparison of the Efficacy and Tolerability of Restylane Versus Zyplast for the Correction of Nasolabial Folds,” Dermatologic Surgery 29, no. 6 (2003): 588–595. 12786700 10.1046/j.1524-4725.2003.29150.x

[13] H. G. Leal Silva , “Facial Laxity Rating Scale Validation Study,” Dermatologic Surgery 42, no. 12 (2016): 1370–1379. 27673484 10.1097/DSS.0000000000000915

[14] M. Amiri , R. Meçani , E. Llanaj , et al., “Calcium Hydroxylapatite (CaHA) and Aesthetic Outcomes: A Systematic Review of Controlled Clinical Trials,” Journal of Clinical Medicine 13, no. 6 (2024): 1686. 38541911 10.3390/jcm13061686 PMC10971119

[15] U. Wollina , C. Wiegand , and U. C. Hipler , “Calcium HYDROXYLAPATITE Microspheres—Biocompatibility and Clinical Effects,” Georgian Medical News 278 (2018): 62–68. 29905547

[16] K. Beer , M. Yohn , and J. L. Cohen , “Evaluation of Injectable CaHA for the Treatment of Mid‐Face Volume Loss,” Journal of Drugs in Dermatology 7, no. 4 (2008): 359–366. 18459517

[17] B. S. F. Bravo , T. S. C. de Almeida , R. d. M. Carvalho , C. J. Machado , L. G. Bravo , and M. C. Elias , “Dermal Thickness Increase and Aesthetic Improvement With Hybrid Product Combining Hyaluronic Acid and Calcium Hydroxyapatite: A Clinical and Sonographic Analysis,” Plastic and Reconstructive Surgery. Global Open 11, no. 6 (2023): e5055. 37334389 10.1097/GOX.0000000000005055 PMC10270556

[18] Y. Alexandrovna Yutskovskaya and A. Kogan , “Improved Neocollagenesis and Skin Mechanical Properties After Injection of Diluted Calcium Hydroxylapatite in the Neck and Décolletage: A Pilot Study,” Journal of Drugs in Dermatology 16, no. 1 (2017): 68–74. 28095536

[19] T. Pavicic , “Calcium Hydroxylapatite Filler: An Overview of Safety and Tolerability,” Journal of Drugs in Dermatology 12, no. 9 (2013): 996–1002. 24002146

